# Scrutinizing pathogenicity of the *USH2A* c.2276 G > T; p.(Cys759Phe) variant

**DOI:** 10.1038/s41525-022-00306-z

**Published:** 2022-06-07

**Authors:** Janine Reurink, Erik de Vrieze, Catherina H. Z. Li, Emma van Berkel, Sanne Broekman, Marco Aben, Theo Peters, Jaap Oostrik, Kornelia Neveling, Hanka Venselaar, Mariana Guimarães Ramos, Christian Gilissen, Galuh D. N. Astuti, Jordi Corominas Galbany, Janneke J. C. van Lith-Verhoeven, Charlotte W. Ockeloen, Lonneke Haer-Wigman, Carel B. Hoyng, Frans P. M. Cremers, Hannie Kremer, Susanne Roosing, Erwin van Wijk

**Affiliations:** 1 Department of Human Genetics, Radboud University Medical Center, Nijmegen, The Netherlands; 2 Donders Institute for Brain Cognition and Behaviour, Radboud University Medical Center, Nijmegen, The Netherlands; 3 Department of Otorhinolaryngology, Radboud University Medical Center, Nijmegen, The Netherlands; 4 Department of Ophthalmology, Radboud University Medical Center, Nijmegen, The Netherlands; 5 Centre for Molecular and Biomolecular Informatics, Radboud Institute for Molecular Life Sciences, Radboud University Medical Center, Nijmegen, The Netherlands; 6 Radboud Institute of Molecular Life Sciences, Radboud University Medical Center, Nijmegen, The Netherlands; 7 Division of Human Genetics, Center for Biomedical Research (CEBIOR), Faculty of Medicine, Diponegoro University, Semarang, Indonesia; 8 Department of Ophthalmology, Elisabeth-TweeSteden Hospital, Tilburg, The Netherlands

**Keywords:** Mutation, Next-generation sequencing, Genetic variation, Animal breeding, Disease genetics

## Abstract

The *USH2A* variant c.2276 G > T (p.(Cys759Phe)) has been described by many authors as a frequent cause of autosomal recessive retinitis pigmentosa (arRP). However, this is in contrast with the description of two asymptomatic individuals homozygous for this variant. We therefore assessed pathogenicity of the *USH2A* c.2276 G > T variant using extensive genetic and functional analyses. Whole genome sequencing and optical genome mapping were performed for three arRP cases homozygous for *USH2A* c.2276 G > T to exclude alternative genetic causes. A minigene splice assay was designed to investigate the effect of c.2276 G > T on pre-mRNA splicing, in presence or absence of the nearby c.2256 T > C variant. Moreover, an *ush2a*^p.(Cys771Phe)^ zebrafish knock-in model mimicking human p.(Cys759Phe) was generated and characterized using functional and immunohistochemical analyses. Besides the homozygous c.2276 G > T *USH2A* variant, no alternative genetic causes were identified. Evaluation of the *ush2a*^p.(Cys771Phe)^ zebrafish model revealed strongly reduced levels of usherin expression at the photoreceptor periciliary membrane, increased levels of rhodopsin localization in the photoreceptor cell body and decreased electroretinogram (ERG) b-wave amplitudes compared to wildtype controls. In conclusion, we confirmed pathogenicity of *USH2A* c.2276 G > T (p.(Cys759Phe)). Consequently, cases homozygous for c.2276 G > T can now receive a definite genetic diagnosis and can be considered eligible for receiving future QR-421a-mediated exon 13 skipping therapy.

## Introduction

Pathogenic variants in *USH2A* explain in general 8-19% of cases with autosomal recessive retinitis pigmentosa (arRP) (OMIM: #613809)^1,2^, 57–90% of Usher syndrome type 2 (USH2) (OMIM: #276901) cases and ~50% of Usher syndrome (USH) cases^3,4^. To date, more than 2,000 unique variants have been reported in *USH2A*, most of which are rare. One of the exceptions is the c.2276 G > T variant (hg19/GRCh37: NM_206933.2; g.216420460 C > A; p.(Cys759Phe)) which was first described by Rivolta et al.^5^. It appeared to be one of the most frequently detected putatively pathogenic variant in *USH2A* and is therefore regarded to be of high clinical significance. The variant was identified both in cases with arRP and in cases with USH2, although it has a higher reported prevalence in arRP (25.4% of *USH2A* variants observed in a *USH2A* cohort) than in USH2 (2.8% of variants)^6^. Further studies confirmed that *USH2A* c.2276 G > T is prevalent, representing ~4.5% of all disease-causing alleles in cohorts of arRP cases^7,8^. In the general population, the overall allele frequency (AF) of c.2276 G > T is 0.097% (273/282,114 alleles, gnomAD v3.1) and 0.14% in the non-Finnish European population (182/128,602 alleles)^9^. No individuals homozygous for the c.2276 G > T variant have been reported in gnomAD (https://gnomad.broadinstitute.org/variant/1-216420460-C-A?dataset=gnomad_r2_1). Also, the variant is classified as pathogenic by the ClinVar expert panel^10^ and was recently reported to be significantly enriched in homozygous state, and in compound heterozygosity with a protein-truncating *USH2A* allele, in a cohort of 982 non-Asian arRP probands after analysis of next-generation sequencing data^11^. These data suggest putative pathogenicity of c.2276 G > T.

The findings described above are in contrast with the description of two unaffected family members of arRP cases (family S23) that were reported to be homozygous for this variant^12^. Extensive genetic testing of family S23 revealed a homozygous variant in *PDE6B* (NM_000283.3: c.1678C > T; p.(Arg560Cys); gnomAD AF: 0.0015%) that fully segregated with arRP in this family. This further strengthened the claim that *USH2A* c.2276 G > T may not be pathogenic and resulted in the recommendation to re-evaluate all families and cases with this variant^13^. Until today, arRP cases that are homozygous for *USH2A* c.2276 G > T therefore do not receive a conclusive genetic diagnosis in a number of diagnostic centers in Europe and the USA. Importantly, a therapeutic approach with antisense oligonucleotides that induce *USH2A* exon 13 skipping (QR-421a) is currently being evaluated in a Phase I/II clinical trial (ClinicalTrials.gov Identifier: NCT03780257). arRP cases carrying the c.2276 G > T variant, which also resides in *USH2A* exon 13, could be potentially eligible for receiving this treatment when reaching the market, in case this variant is proven to be pathogenic^14^.

With the aim to affirm pathogenicity of the c.2276 G > T (p.(Cys759Phe)) variant in *USH2A*, we implemented a comprehensive array of genetic and functional tests including whole genome sequencing (WGS) for three arRP cases homozygous for the *USH2A* c.2276 G > T variant and optical genome mapping (OGM) in two of these cases to exclude hidden structural variants. A minigene splicing assay was performed to determine a potential effect of this variant on *USH2A* pre-mRNA splicing, as well as molecular modeling of the effect of the p.(Cys759Phe) variant on the structure of the associated protein domain. Furthermore, we confirmed a pathogenic effect through a thorough phenotypic assessment of a generated zebrafish knock-in model, *ush2a*^*p.(Cys771Phe)*^, that mimics the human *USH2A* c.2276 G > T (p.(Cys759Phe)) variant.

## Results

### Whole genome sequencing of arRP cases homozygous for *USH2A* c.2276 G > T does not reveal additional pathogenic variants

WGS was performed for three individuals with arRP (cases I, II and III), that previously underwent diagnostic exome sequencing and were shown to be homozygous for the *USH2A* c.2276 G > T variant. To exclude other causes of arRP in these cases, such as intronic variants, structural variants and variants in regulatory elements, a total of 14,343 exonic and intronic single nucleotide variants (SNVs) were evaluated in all 63 previously published arRP-associated genes^15^. Of these SNVs, 1187 have an AF ≤ 1% in gnomAD and our in-house exome sequencing database containing variants identified in 24,488 individuals. No (likely) pathogenic homozygous or compound heterozygous SNVs were observed, with the exception of *USH2A* c.2276 G > T (Supplementary Table 1).

All SNVs in *USH2A* and 200 kilobases upstream and downstream of *USH2A* (*n* = 1615) were extracted from WGS data and the number of alleles carrying each SNV was extracted. From chr1:216632415 (35 kb upstream of *USH2A*) to chr1:216247667 (*USH2A* intron 27), the majority of SNVs are shared amongst all alleles in the three cases, indicating a shared haplotype (Supplementary Fig 1). SNVs between chr1:216241286 (*USH2A* intron 30) and chr1:216211989 (*USH2A* intron 32) are shared amongst four alleles. In total, 1117 SNVs within the genomic region of *USH2A* were evaluated. Seven SNVs have an AF ≤ 1% and are shared amongst all three cases and were further investigated as potential variants of interest (Supplementary Table 2). Of these seven variants, c.2276 G > T, c.784 + 9428 A > G and c.4628-23020_4628-23007del were identified in a homozygous state in all cases, whereas the synonymous variant c.2256 T > C (p.(His752 = ), gnomAD AF: 0.071%) was homozygous in cases I and III and heterozygous in case II. None of the intronic variants, either shared amongst all cases or unique, identified in *USH2A* were predicted to have an effect on splicing (>0.1) upon assessment using SpliceAI prediction software^16^ or were predicted to have a strength of 75% and to result in a minimal increase of 2% in strength for two of the following algorithms: SpliceSiteFinder-like^17^, MaxEntScan^18^, NNSPLICE^19^ and GeneSplicer^20^, as previously established^21^. All variants were therefore considered irrelevant.

Potential *USH2A* regulatory elements were determined based on a database containing predicted *cis*-regulatory elements identified in post-mortem human retina tissue by Cherry et al.^22^, epigenetic data derived from mouse photoreceptor cells^23^, data on open chromatin structures derived from the mouse inner ear^24^ and DNA-methylation data derived from mice cochlear sensory epithelium^25^. The Cherry and Genehancer databases were employed to assess putative enhancers that associate with the putative *USH2A* promoter region^22,26^. The ten most prominent regions that were identified in at least two out of these five databases, including the predicted promoter region, were selected (Supplementary Table 3). Seven, mainly homozygous and shared, SNVs are present in these ten selected potential regulatory regions of *USH2A* (Supplementary Table 4). None of these variants has an AF ≤ 1% and these variants are therefore considered benign.

Eight heterozygous variants with an AF < 1% were detected within *PDZD7*, a previously described modifier for *USH2A*-associated retinal disease^27^ (Supplementary Table 5). None of the variants were shared amongst cases. Seven variants were intronic or synonymous, were not predicted to have an effect on splicing, and therefore were considered benign. One variant is located in the 3’UTR and has an AF of 1.04% in the non-Finnish European population. This variant is located in a G-stretch and therefore unlikely to have any effect.

### Variants c.2276 G > T and c.2256 T > C do not affect *USH2A* pre-mRNA splicing

**Fig. 1:**
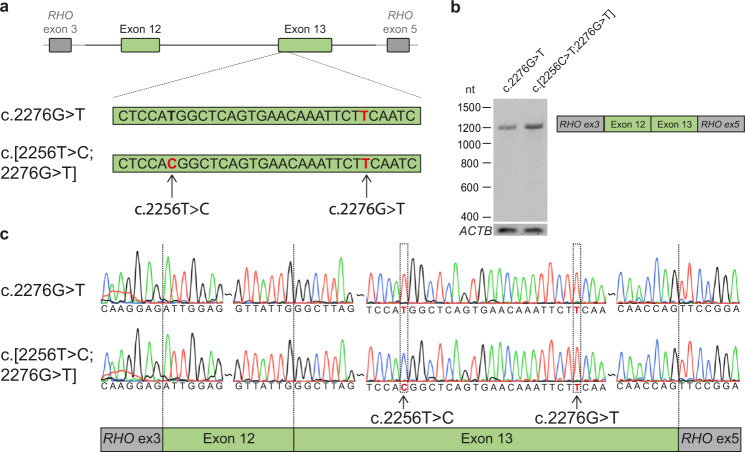
Minigene splice assays for variants c.2256 C > T and c.2276 G > T. **a** A minigene splice assay was performed with a construct spanning from *USH2A* intron 11 to intron 13 (6,814 nt), containing either c.2276 G > T or both variants (c.[2256 T > C;2276 G > T]). **b** A single RT-PCR product of 1112 nt was observed after expression of both splice vectors, indicative of the incorporation of *USH2A* exons 12 and 13 between *RHO* exons 3 and 5 in both transcripts. **c** Sanger sequencing confirmed that *USH2A* exon 12 and exon 13 were correctly incorporated in the mRNA.

Although c.2276 T > C was not predicted to have an effect on splicing, we wanted to address such an effect. Therefore, we performed minigene splice assays in HEK293T cells to assess a potential effect of the variant on *USH2A* pre-mRNA splicing either alone or in combination with the nearby synonymous variant c.2256 T > C (p.(His752 = )) present in *cis* (Fig. 1). The c.2256 T > C variant was already shown to reside in *cis* with the c.2276 G > T variant when it was first reported^5^. No indications for alternative splicing were observed and Sanger sequencing confirmed that both exons 12 and 13 were correctly incorporated in the mRNA. Therefore, a pathogenic effect on *USH2A* pre-mRNA splicing resulting from c.2276 G > T and from the complex allele c.[2256 T > C;2276 G > T] was excluded in HEK293T cells.

### No pathogenic structural variants were identified in arRP-associated genes

The three studied cases were assessed for the presence of putatively pathogenic structural variants (SVs) or copy number variants (CNVs) in 63 arRP-associated genes, including *USH2A*. In case I, a large inversion was detected encompassing the entire *USH2A* gene. However, the breakpoints are located more than 18 Mb up- and downstream of the *USH2A* gene and its predicted regulatory regions. Moreover, this SV was found in 43% of the Wellderly population database (https://ega-archive.org/studies/EGAS00001002306/) containing 1022 alleles. Therefore, a putative pathogenic or modifying effect on *USH2A* expression was considered highly unlikely. Five heterozygous deletions encompassing known arRP-associated genes were identified. The regions of these deletions were manually inspected using the Integrative Genomics Viewer software (v2.4.11). In all five regions that were predicted to be heterozygously deleted, several heterozygous SNVs were identified, indicating the presence of two alleles. Therefore, these deletions are likely false positive calls present in the WGS data.

To enable a more accurate detection of chromosomal aberrations that potentially could have been missed by our short-read WGS approach, we employed OGM for case II and III. A total of 6761 and 6739 possible SVs were detected in cases II and III, respectively. Evaluation of these SVs did not reveal any rare (<1% AF in control samples) or unique SVs within or in the vicinity of genes associated with arRP.

### Modeling the structural effect of the p.(Cys759Phe) variant on laminin–epidermal growth factor domain 5

The p.(Cys759Phe) variant affects the third cysteine residue within the fifth of ten consecutive laminin-epidermal-growth-factor (EGF Lam) domains that are predicted to be present in usherin^28^. EGF Lam domains typically contain eight cysteine residues that interact in a pairwise fashion (Cys1 + Cys3; Cys2 + Cys4; Cys5 + Cys6; Cys7 + Cys8) to form four covalent disulfide bonds, that are essential for protein folding and stability. Twenty-nine of the 80 cysteine residues in the usherin EGF Lam domains have been reported to be mutated (*USH2A* LOVD variation database, https://www.lovd.nl/USH2A; consulted July 13, 2021), indicating that these residues are crucial for usherin function. We assessed the effect of the p.(Cys759Phe) substitution on the structure of EGF Lam domain 5 by molecular modeling (Supplementary Fig 2). The loss of a covalent cysteine bond is expected to cause local destabilization of the domain structure. Furthermore, Cys747 is present as an unpaired cysteine residue containing a reactive-free thiol group that can induce unwanted multimerization or crosslinking with other proteins^29^. Based on this model, we consider the p.(Cys759Phe) variant detrimental for usherin folding and function.

### Functional assessment of the p.(Cys759Phe) variant in an *ush2a* zebrafish knock-in model

**Fig. 2:**
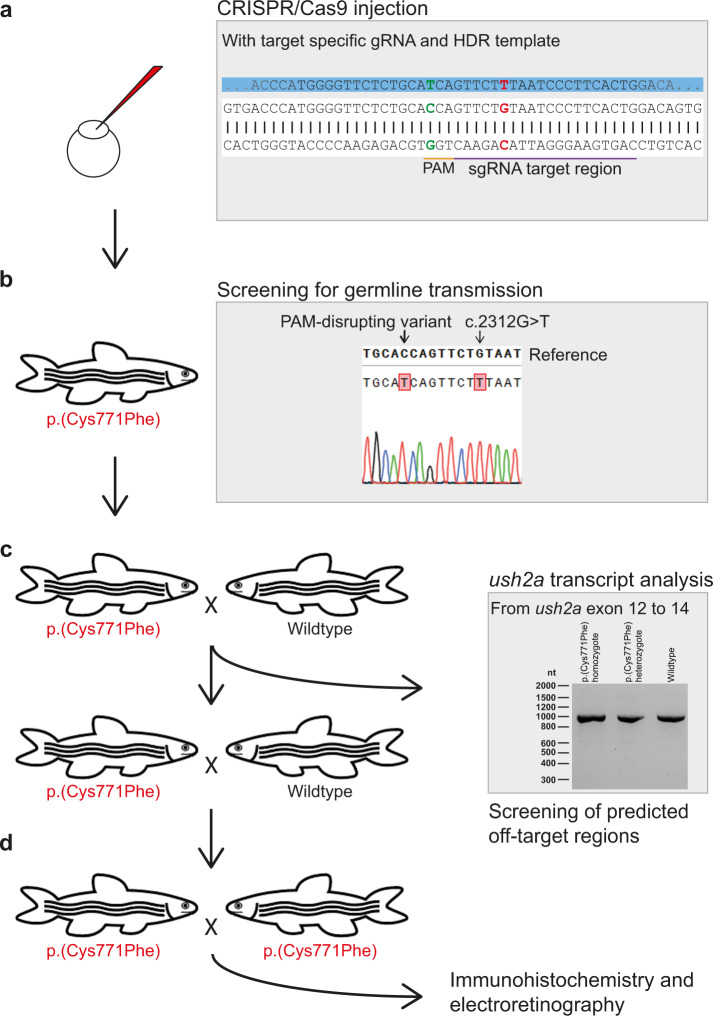
Generation of the usherin^p.(Cys771Phe)^ zebrafish model. **a** Eggs were injected at a one-cell stage with CRISPR/Cas9 mix containing both the target specific single guide RNA (sgRNA) and the 126 nt homology directed repair (HDR) template (partially depicted, in blue) for incorporation of the c.2312 G > T (p.(Cys771Phe)) variant (in red) and protospacer-adjacent motif (PAM, in orange) disrupting variant (c.2304 C > T, in green). The sgRNA target region is depicted in purple. **b** Once zebrafish were at reproductive age, eggs of the initially injected zebrafish were screened for transmission of the c.2312 G > T and PAM disturbing variant. Three out of ten zebrafish that were screened transmitted the c.2312 G > T variant to their offspring. **c** Crossbreeding of a c.2312 G > T (p.(Cys771Phe)) zebrafish with a wildtype zebrafish was performed for two generations to reduce unforeseen off-target effects. After the first crossbreeding, genomic DNA was screened for predicted off-target effects of our CRISPR-Cas9 strategy and RNA of homozygous larvae was screened from exon 12 to 14 for deviations on transcript level. **d** Two p.(Cys771Phe) zebrafish were crossbred with each other to produce homozygous zebrafish. The phenotype of five-day-old larvae was then investigated with immunohistochemistry and electroretinography.

In order to assess the effect of the usherin p.(Cys759Phe) variant on visual function, we used CRISPR/Cas9 technology to generate a zebrafish p.(Cys771Phe) knock-in model, which is the equivalent of the human usherin p.(Cys759Phe) variant. Zebrafish has previously been shown to be a relevant model for *USH2A*-associated arRP^30,31^. Fertilized eggs (*n* = 197) at a single-cell stage were injected with a CRISPR/Cas9 mixture consisting of Cas9 endonuclease and a target specific sgRNA (Fig. 2a). In addition, a 126 nt oligonucleotide homology directed DNA-repair (HDR) template was included in the mixture to enable incorporation of the c.2312 G > T variant (p.(Cys771Phe); ENSDART00000086201.4). The HDR template furthermore contains a silent variant (c.2304 C > T; p.(His768 = )) that disrupts the protospacer-adjacent motif (PAM) and prevents repeated Cas9 cleavage after introduction of c.2312 G > T.

The offspring (F1) of ten individual adult F0 zebrafish was screened for germline transmission of variant c.2312 G > T after cross-breeding with strain-matched wildtypes^32^. Three out of ten screened F0 zebrafish successfully transmitted the variant to their progeny. We did not detect any alterations in *ush2a* exon 13 other than c.2312 G > T and c.2304 C > T, compared to DNA from wildtype zebrafish (Supplementary Fig 3). In order to minimize the presence of potential CRISPR/Cas9-induced off-target modifications, two generations of outbreeding with strain-matched wildtypes were performed (F2), followed by an inbreeding of two heterozygous zebrafish. The resulting model is homozygous for both the variant of interest (c.2312 G > T; p.(Cys771Phe)) and the silent PAM-disrupting variant (c.2304 C > T; p.(His768 = )) (Fig. 2b). Transcript analysis on homozygous c.2312 G > T larvae was performed to identify potential effects on *ush2a* pre-mRNA splicing resulting from the introduction of the *ush2a* c.2312 G > T and c.2304 C > T. RT-PCR from exons 12 to 14 on larval mRNA of amplicons did not reveal any alternative splicing events or other sequence alterations in homozygous knock-in zebrafish as compared to their wildtype siblings, indicating that both introduced in *ush2a* exon 13 do not have an effect on splicing (Fig. 2c), which was validated by Sanger sequencing.

Four potential off-target regions for the used sgRNA were identified within the zebrafish genome using the Cas-OFFinder webtool^33^. F2 larvae derived from either of the three germline-positive F0 founder zebrafish (fish 1–3) were screened for the presence of potential off-target edits by PCR and Sanger sequencing (Supplementary Table 6). One SNV was identified (heterozygous in fish 1 and homozygous in fish 2 and 3) in the vicinity of the predicted off-target region on chromosome 20 (chr20:41755382 G > A (GRCz10)). However, the variant was located 86 nt upstream of the predicted off-target site and labeled as a known SNV in the ENSEMBL zebrafish genome browser. The SNV was therefore considered irrelevant in our screening. A second SNV (chr5:40167334 G > A (GRCz10)) was identified exactly at the predicted off-target site in heterozygous state in fish 1. We therefore decided to exclude this zebrafish from further studies. For a predicted off-target site on chromosome 17 (chr17:25767450 (GRCz10)), no sequence aberrations were observed. We failed to amplify the region of interest for a fourth potential off-target site located deep within intron3 of the *myo18b* gene (KX421389) at chromosome 10 (chr10:44374669 (GRCz10)).

### Introduction of p.(Cys771Phe) results in reduced usherin expression levels, pronounced rhodopsin mislocalization and reduced ERG traces

**Fig. 3:**
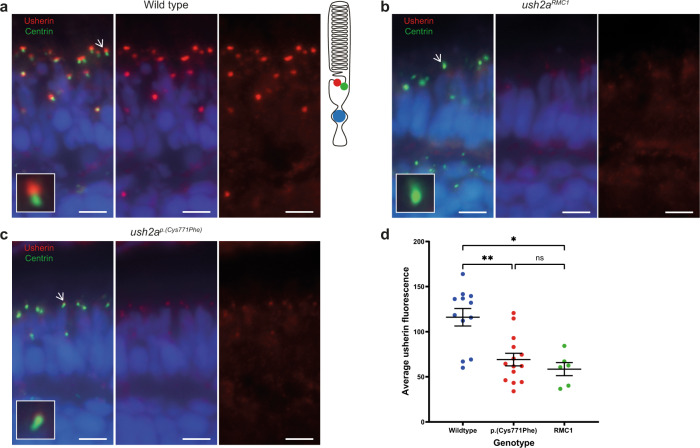
Reduced expression level of usherin^p.(Cys771Phe)^ at the photoreceptor periciliary membrane. **a** In wildtype zebrafish larval eyes (*n* = 12 eyes), usherin (red signal) localizes at the photoreceptor periciliary membrane adjacent to the basal body and connecting cilium marker centrin (green signal) as shown by the schematic representation of a photoreceptor on the right. A magnification of one photoreceptor (indicated by an arrow) is depicted in the inlay. **b** In *ush2a*^*rmc1*^ knock-out larvae usherin is not detectable (*n* = 6 eyes). **c** Localization of usherin at the photoreceptor periciliary membrane was strongly reduced in eyes of *ush2a*^*p.(Cys771Phe)*^ larvae (*n* = 14 eyes) as compared to wildtype. **d** A Kruskal–Wallis test was performed based on the average of the mean grey value for usherin adjacent to each centrin spot and confirmed a significant decrease of usherin localization adjacent to centrin for the usherin^p.(Cys771Phe)^ and the usherin^RMC1^ models. The average grey value per retinal section was plotted in a scatter plot (mean ± SEM). Nuclei are stained with DAPI (blue signal). Scale bar: 5 µM. **p: 0.0094, *p: 0.0107, ns not significant.

To detect functional effects of the p.(Cys771Phe) substitution, we evaluated the expression and subcellular localization of usherin using cryosections of *ush2a*^*p.(Cys771Phe)*^ larval eyes (5 days post-fertilization (dpf); *n* = 14 eyes of 7 larvae), as well as of strain- and age-matched wildtypes (*n* = 12 eyes of 6 larvae) and usherin knock-outs (*ush2a*^*rmc1*^) (*n* = 6 eyes of 3 larvae). As was previously described^30^, usherin localizes to the photoreceptor periciliary membrane adjacent to the basal body and connecting cilium marker centrin in wildtype larvae (Fig. 3a), and is absent from retinal cryosections of *ush2a*^*rmc1*^ knock-out larvae (Fig. 3b). In our *ush2a*^*p.(Cys771Phe)*^ mutants, the usherin signal intensity at the periciliary membrane appeared significantly decreased and no clear mislocalization of mutant usherin protein at other regions within the larval zebrafish retina was observed (Fig. 3c). These results suggest that the p.(Cys771Phe) variant significantly reduces usherin expression in the zebrafish photoreceptors (Fig. 3d).

**Fig. 4:**
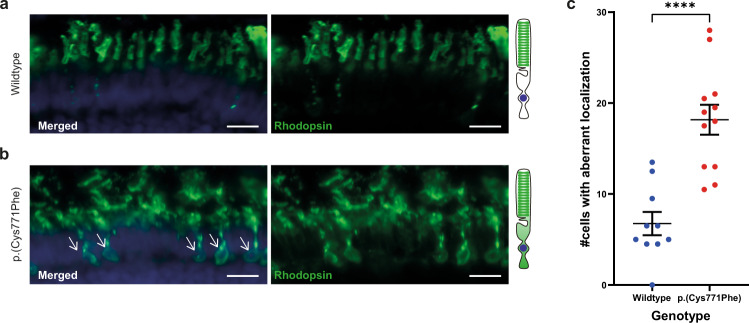
Rhodopsin localization to the photoreceptor cell body in the *ush2a*^*p.(Cys771Phe)*^ zebrafish. **a** Rhodopsin (green signal) localizes to the rod photoreceptor outer segments in wildtype larval eyes. A visual representation of a photoreceptor is shown on the right. **b** Aberrant localization of rhodopsin to the photoreceptor cell body was observed in *ush2a*^*p.(Cys771Phe)*^ larvae (indicated by white arrows), which was observed in a significantly higher number of photoreceptors of *ush2a*^*p.(Cys771Phe)*^ larvae as compared to wildtype larvae (**c**; unpaired *t* test). Nuclei are stained with DAPI (blue signal). Scale bar: 10 µM. ****p < 0.0001.

As rhodopsin was previously found to be partially mislocalized towards the photoreceptor cell body of *ush2a* zebrafish knock-out models^31^, we also investigated the localization of rhodopsin in our *ush2a*^*p.(Cys771Phe)*^ knock-in model. Cryosections were made from wildtype (*n* = 10 eyes of 5 larvae) and *ush2a*^*p.(Cys771Phe)*^ larval eyes (*n* = 12 eyes of 6 larvae). We observed a significant increase in the number of photoreceptor cells with aberrant rhodopsin localization in *ush2a*^*p.(Cys771Phe)*^ as compared to matched wildtype controls (unpaired Student’s *t* test (two-tailed); *p* < 0.0001; t: 5.318, df: 20) (Fig. 4).

**Fig. 5:**
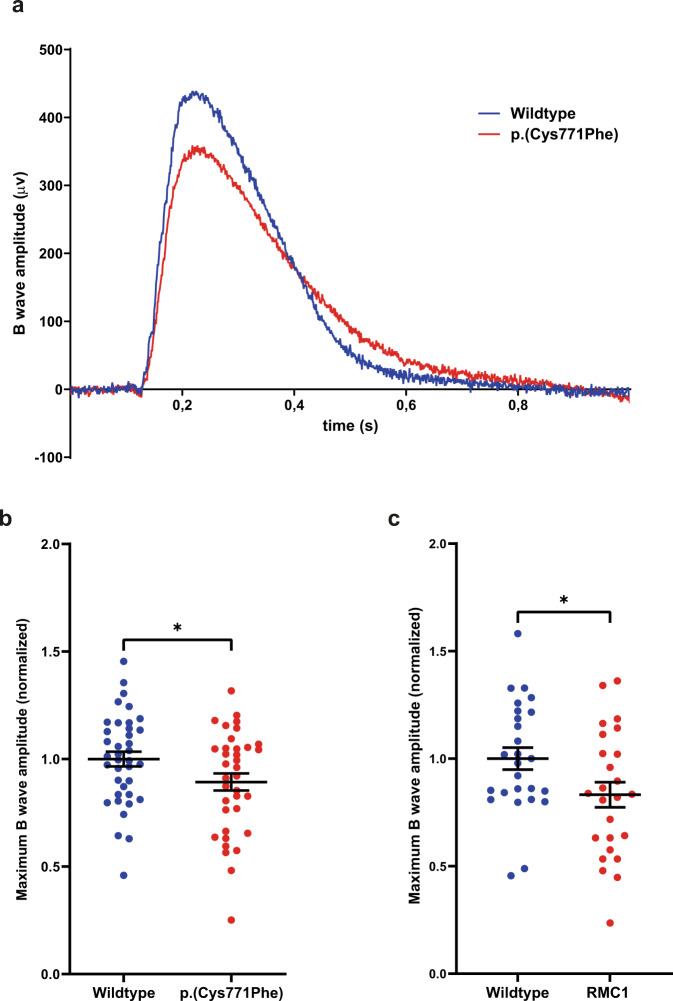
Electroretinogram recordings show that *ush2a*^*p.(Cys771Phe)*^ mutants are vision impaired. **a** Representative electroretinograms of a p.(Cys771Phe) zebrafish and a wildtype sibling at 5 days post-fertilization. **b** The maximum B wave amplitude was significantly lower in p.(Cys771Phe) zebrafish as compared to wildtype siblings (unpaired *t* test). The average wildtype amplitude was normalized to 1. Each datapoint corresponds to recordings from an individual larvae (mean ± SEM). *p: 0.0445. **c** A comparative analysis of *ush2a*^*rmc1*^ knock-out larvae and age- and strain-matched wildtype larvae was shown to result in a similar and significant decrease in maximum B wave amplitude. Again, the average wildtype amplitude was normalized to 1. Each datapoint corresponds to recordings from an individual larvae (unpaired *t* test, mean ± SEM). *p: 0.0345.

Finally, ERG responses were recorded for both wildtype (*n* = 38 from three biological replicates) and p.(Cys771Phe) (*n* = 36 from three biological replicates) larvae at 5 dpf. A significant decrease in maximum B wave amplitudes (~11%) was observed in the p.(Cys771Phe) larvae as compared to wildtype larvae (unpaired Students *t* test (two-tailed); *p*: 0.0445; t: 2.045; df: 72), which is indicative of an impaired visual function (Fig. 5b). ERG responses were also recorded for *ush2a*^*rmc1*^ knock-out larvae (5 dpf; *n* = 25 from three biological replicates) and age- and strain-matched wildtype siblings (*n* = 26 from three biological replicates). In the knock-out model a similar significant decrease in maximum B wave amplitude of ~15% compared to age- and strain-matched wildtype larvae was observed (unpaired Student’s *t* test (two-tailed); *p*: 0.0345; t: 2.175, df: 49) (Fig. 5c).

## Discussion

Here, we confirmed the pathogenicity of the *USH2A* c.2276 G > T (p.(Cys759Phe)) variant, based on comprehensive genetic analyses, protein modeling and functional assessment of a zebrafish knock-in model. We did not identify any other potentially pathogenic variants within the haplotype containing c.2276 G > T, neither did we identify bi-allelic variants in other arRP-associated genes. With protein modeling we showed that the substitution of phenylalanine for cysteine affects one of four disulfide bridges essential for a proper folding of EGF Lam domain 5, which is indicative of the pathogenic nature of p.(Cys759Phe). Finally, we evaluated the effect of the p.(Cys759Phe) variant on visual function after scrutinizing the *ush2a*^*p.(Cys771Phe)*^ zebrafish knock-in model. We observed significantly reduced levels of usherin p.(Cys771Phe) at the zebrafish photoreceptor periciliary membrane and increased levels of aberrantly localized rhodopsin, which was previously shown to be a hallmark of *ush2a*-associated retinopathy in a zebrafish knock-out model^31^. Moreover, ERG recordings demonstrated that visual function was impaired in *ush2a*^*p.(Cys771Phe)*^ zebrafish larvae. These results are similar to the reduced B wave amplitudes recorded in the previously published *ush2a*^*rmc1*^ knock-out model (Fig. 5c) and similar to the previously published ERG recordings on *ush2a* knock-out zebrafish models shown by Han et al. and Toms et al., confirming pathogenicity of the p.(Cys771Phe) variant^31,34^.

Our conclusion that c.2276 G > T is pathogenic is in agreement with the analyses provided by many other groups, but does not correspond with the conclusion of Bernal et al^12^. In their paper, they reported two healthy individuals of family S23 that are homozygous for c.2276 G > T. As a result, Bernal et al. recommended a genetic re-evaluation of families with this variant. A panel of 26 IRD-associated genes was subsequently analyzed and the absence of the deep-intronic c.7595–2144 A > G variant in *USH2A* was verified by Sanger sequencing in affected and unaffected individuals of family S23^13^. With this approach, a homozygous variant in *PDE6B* (c.1678C > T (p.(Arg560Cys)) was identified in affected family members. The variant is equivalent to mouse *Pde6b* c.1678C > T (p.(Arg560Cys), which has been identified as the causal variant in a previously reported mouse model for retinal degeneration^35^. In contrast, the healthy homozygous carriers of *USH2A* c.2276 G > T were shown to be heterozygous for the variant in *PDE6B*. Currently, we can only speculate about a possible explanation for the discrepancy between our findings and the findings of Bernal and colleagues. Reanalysis of family S23 with current genetic screening methods, such as WGS and OGM, would be recommendable. A first hypothesis could be an undetected and novel (deep) intronic variant resulting in the in-frame excision of *USH2A* exon 13 in these individuals. Skipping of specifically *USH2A* exon 13 from the transcript was recently shown to result in the production of a functional usherin protein^14^. A second hypothesis is the presence of modifier variants. *PDZD7* is a known disease-aggravating modifier of *USH2A*-associated retinal disease^27^. However, to date no protective modifiers of *USH2A*-associated disease have been reported, in contrast to what has been reported for other disorders. For example, a homozygous deletion of the *SMN1* gene results in a spinal muscular atrophy (SMA) phenotype, however asymptomatic individuals have also been reported having the same homozygous deletion as their affected siblings^36^. Unaffected individuals exhibit increased levels of PLS3 which rescues the detrimental effects on axon growth that underly SMA and as a consequence *PLS3* was suggested to act as a protective modifier for SMA. Also in sensory disorders, a similar protective effect has been observed in a mouse model for *GRXCR2-*associated hearing loss^37^. *Grxcr2*-deficient mice showed pronounced hearing loss and disorganized stereocilia. However, reduced levels of taperin, which has also been linked to deafness, in *Grxcr2*-deficient mice restored their hearing and stereocilia morphology.

Although not identified so far, protective modifiers of *USH2A*-associated disease might exist and similar mechanisms as described for SMA and deafness could protect against *USH2A* c.2276 G > T-associated arRP. Either a negative modifier or an unknown pathogenic variant on the c.2276 G > T haplotype could result in disease in the majority of cases with c.2276 G > T-associated arRP, but these could be absent in family S23. DuPont and colleagues identified a shared haplotype of 199 kilobases, spanning from *USH2A* exon 14 to exon 25, that could harbor a pathogenic variant other than c.2276 G > T^11^. Based on our WGS data, we also determined a haplotype by identifying SNVs that are shared amongst all three cases in our study (Supplementary Fig 1). A ‘homozygosity block’ was observed with SNVs that are shared amongst all alleles and that spans the region from 35 kb upstream of *USH2A* to intron 27. This supports the conclusion from DuPont and co-workers of a shared haplotype containing c.2276 G > T. However, they suggest a possible recombination causing c.2276 G > T to be no longer linked with the true disease-causing variant in the family of Bernal et al. 2003. To the best of our ability, we excluded the presence of pathogenic variants other than c.2276 G > T in the cases in our study with both WGS and OGM, and demonstrated that a possible recombination is highly unlikely.

Our study provides indisputable evidence that the *USH2A* c.2276 G > T; p.(Cys759Phe) variant is pathogenic, which can be further demonstrated in the future by treatment of arRP cases homozygous for *USH2A* c.2276 G > T with QR-421a^14^. Future studies will be needed to unravel the exact mechanism of disease underlying arRP caused by the p.(Cys759Phe) variant in usherin.

## Methods

### Whole genome sequencing

DNA of three unrelated probands (I, II and III), clinically diagnosed with arRP and homozygous for c.2276 G > T, was obtained by the Radboud University Medical Center (Nijmegen, the Netherlands). Written informed consent was received from all individuals, adherent to the tenets of the Declaration of Helsinki and as approved by the local ethics committee of the Radboud University Medical Center Nijmegen, as an amendment to the approval by the local ethics committee of the Rotterdam Eye Hospital (MEC-2010-359; OZR protocol no. 2009-32). The clinical status of all cases is listed in Supplementary Table 7. WGS data processing and variant calling was performed using genome assembly 37 (hg19), as published previously^38^.

WGS data of the three cases were evaluated for variants in 63 genes described to be associated with arRP (Supplementary Table 8) (Retnet; visited May 21, 2021)^15^, *PDZD7*^27^ and potential *USH2A* regulatory regions^22–26^. SNVs were considered potentially pathogenic if they had an overall gnomAD AF ≤ 1%^9^ and were either (1) a stop gain variant, frameshift variant or canonical splice site variant, (2) a missense variant with either; Grantham score ≥ 80 (range: 5–215), CADD_PHRED ≥ 15 (range: 0–99) or PhyloP ≥ 2.7 or 3) a putative splice-modulating variant with a spliceAI score ≥ 0.1 (default settings, range 0–1)^16^ or a score ≥ 75% with a minimal increase of 2% in strength^21^ for two of the following algorithms: SpliceSiteFinder-like^17^, MaxEntScan^18^, NNSPLICE^19^ and GeneSplicer^20^.

CNVs and SVs that passed our quality filter and had a high quality score (≥500/1000) were prioritized if these affect the aforementioned 63 genes associated with arRP. OGM was performed for cases II and III, as described previously^39,40^. Variants with an AF ≤ 1% in 107 control samples and that overlap genes that are known to be involved in arRP (Supplementary Table 8) were evaluated.

### Minigene splice assay

*USH2A* exons 12 and 13 and ~1000 nt of intronic sequence upstream of exon 12 and downstream of exon 13 were amplified from genomic DNA of case II (heterozygous for c.2256 T > C (NM_206933.2; p.(His752 = )), homozygous for c.2276 G > T) (primers 5’-GGGGACAAGTTTGTACAAAAAAGCAGGCTTCcctccaaatgtgaagagctgg-3’(forward); 5’-GGGGACCACTTTGTACAAGAAAGCTGGGTGtgcagtggtccttctccttag-3’(reverse)). Both c.2276 G > T and c.[2256 T > C;2276 G > T] fragments were individually cloned between *RHO* exons 3 and 5 of an adapted pCI-NEO vector, with Gateway^®^ cloning technology (Thermo Fisher Scientific, Carlsbad (CA), USA), as described previoiusly^41^. *USH2A* cDNA was amplified (primers 5’-CGGAGGTCAACAACGAGTCT-3’(forward); 5’-AGGTGTAGGGGATGGGAGAC-3’(reverse)) as well as *ACTB* cDNA as loading control (primers 5’-ACTGGGACGACATGGAGAAG-3’(forward); 5’-TCTCAGCTGTGGTGGTGAAG-3’(reverse)). Both samples and loading control were processed in parallel.

### Modeling the fifth laminin–epidermal growth factor like domain

A homology model of the fifth EGF Lam domain was created using the YASARA & WHAT IF Twinset homology modeling module^42,43^. A hybrid model of residues 747–792 (PDB files 5LF2) was created representing Laminin beta 2 from the rat, with a sequence identity of 46%^44^.

### Zebrafish maintenance and husbandry

The animal experiments were approved by the Radboud University Institutional Review Board of the Centrale Commissie Dierproeven (AVD103002017945). All experiments were carried out in accordance with European guidelines on animal experiments (2010/63/EU). Wildtype Tüpfel Long fin (TLF) zebrafish were used. All animals were maintained and raised by standard methods^45^.

### CRISPR/Cas9 approach for the generation of an *ush2a*^*p.(Cys771Phe)*^ knock-in zebrafish model

A constant single guide RNA (sgRNA-1, AAAAGCACCGACTCGGTGCCACTTTTTCAAGTTGATAACGGACTAGCCTTATTTTAACTTGCTATTTCTAGCTCTAAAAC), a target specific single guide RNA (sgRNA-2, CCGCTAGCTAATACGACTCACTATAGAGTGAAGGGATTACAGAACGTTTTAGAGCTAGAAATAGCAAG) and a 126 nt oligonucleotide template (TTGTAACTATGGCTTCAAATTCCTCAATCACACCAATCCCGATGGTTGCATTTCCTGTGGCTGTGACCCATGGGGTTCTCTGCATCAGTTCTTTAATCCCTTCACTGGACAGTGTGAGTGCAAAGC) for HDR were designed, generated and micro-injected in zebrafish zygotes as previously described^32^. The resulting *ush2a*^*p.(Cys771Phe)*^ allele is deposited as *ush2a*^*rmc016*^ in the ZFIN database (www.zfin.org).

### Genotyping

Larvae were collected from separate breeding pairs 1 dpf. Zebrafish samples, being either individual embryos (1 dpf), larval tail fins (5 dpf) or adult tail fins, were lysed in 25 µl (embryos, larval tails) or 75 µl (adult tail fins) lysis buffer (0.2 mM EDTA, 40 mM NaOH) at 95 °C for 20 min and then neutralized with 2.5 µl or 7.5 µl 1 M Tris-HCl (pH 8). Obtain DNA was used as input for a PCR using standard cycling conditions to amplify the genomic region of *ush2a* exon 13 (primers 5’-TTCCTCAATCACACCAATCCCG-3’(forward); 5’-TGCCTCTGAGATCACACTGA-3’(reverse)). Fragments were analyzed using Sanger sequencing.

### Screening for CRISPR/Cas9-induced off-target editing

Cas-OFFinder was employed to identify potential off-target regions for the used sgRNA, using cut-off values of <3 mismatches and <2 bulges^33^. A genomic PCR was performed to amplify all identified regions (Supplementary Table 6), and amplicons were screened with Sanger sequencing.

### Transcript analysis

Total RNA was isolated from larval heads of an F1-incross using the RNeasy Micro kit according to manufacturer’s instructions (Qiagen, Hilden, Germany). 200 µg of total RNA was used for cDNA synthesis with the SuperScript IV Reverse Transcriptase kit (Thermo Fisher Scientific, Waltham (MA), USA). PCR was performed on cDNA according to standard protocol, from *ush2a* exon 12 to exon 14 (primers are listed in Supplementary Table 6), followed by Sanger sequencing.

### Immunohistochemistry

Cryosections (7 µM) of unfixed larvae (5 dpf) were treated, stained and imaged as described previously^14,46^ with primary antibodies directed against zebrafish usherin (rabbit anti-usherin-C (1:500; #27640002; Novus Biological, Centennial (CO), USA) and centrin (mouse anti-centrin (1:500; #04-1624; Millipore, Burlington (MA), USA)). The following secondary antibodies were used: goat anti-rabbit Alexa Fluor^®^ 568 (1:800, A11011, Molecular Probes, Eugene (OR), USA) and goat anti-mouse Alexa Fluor^®^ 488 (1:800, A11029, Molecular Probes, Eugene (OR), USA) and DAPI (1:800, D1306; Molecular Probes, Eugene (OR), USA).

For rhodopsin, cryosections (7 µM) of PFA 4% fixed larvae (5 dpf) treated, stained and imaged as published previously^31,47^. Primary antibody: mouse anti-rhodopsin (1:2000, Clone 4D2; NBP2-59690, Novus Biological, Centennial (CO), USA), goat anti-mouse Alexa Fluor^®^ 488 (1:800, AA11029, Molecular Probes, Eugene (OR), USA) and DAPI (1:800, D1306; Molecular Probes, Eugene (OR), USA).

### Quantification of usherin and rhodopsin localization

Usherin localization at the photoreceptor periciliary membrane was quantified using an automated Fiji (v.1.51n) script as previously published^30^. Rhodopsin mislocalization was quantified by manually scoring the number of photoreceptor cells per retinal cryosection with clear rhodopsin localization in the photoreceptor cell body. All images were blinded, randomized and scored independently by two researchers.

### Electroretinograms

ERG recordings were performed on isolated larval eyes (5 dpf) as previously described^30^. Larvae were dark-adapted for at least 30 min prior to testing. Isolated eyes were stimulated with a light pulse with an intensity of 6000 lux. Electrical signals were captured and subsequently amplified using an electrode that was positioned at the center of the cornea.

### Statistical analysis

All statistical analyses were performed using PRISM software (v9.0.0). Average scores were calculated and normality was checked, followed by an unpaired two-tailed Student’s *t* test (rhodopsin localization assay and ERG recordings) or a Kriskal–Wallis test followed by a Dunn’s multiple comparison test (usherin quantification).

### Reporting summary

Further information on research design is available in the Nature Research Reporting Summary linked to this article.

## Supplementary information

Supplemental material

Reporting Summary Checklist

## Data Availability

Data are available upon request. All whole genome sequencing variants that were considered to be potentially pathogenic are available in the supplementary data. Pathogenic variant data are uploaded to the Leiden Open Variation Database (https://databases.lovd.nl/shared/genes/USH2A). All other whole genome sequencing data are subject to controlled access because these may compromise research participant privacy. These data may become available upon a data transfer agreement approved by the local ethics committee and can be obtained from corresponding author S.R. upon reasonable request.
