# Supplementary material for: Scrutinizing pathogenicity of the USH2A c.2276 G > T; p.(Cys759Phe) variant
Source: NPJ Genom Med. 2022 Jun 7;7:37. doi: 10.1038/s41525-022-00306-z (PMC9174243; doi:10.1038/s41525-022-00306-z)
Supplement: Supplementary file 1 — Supplemental material [file 41525_2022_306_MOESM1_ESM.pdf]

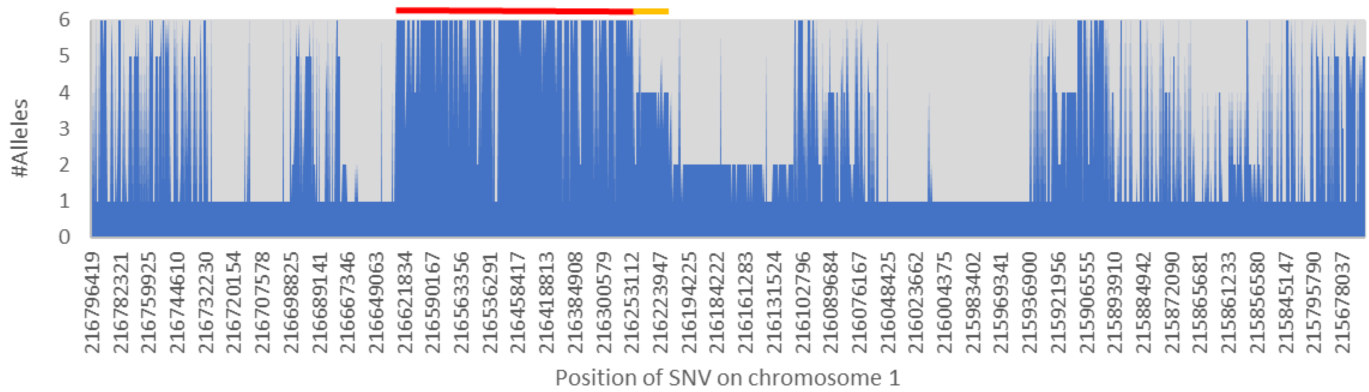

Supplementary Figure 1: Identification of a shared haplotype block associated with the *USH2A* c.2276G>T variant. All single nucleotide variants (SNVs, n=1,615) in *USH2A* and 200 kilobases of flanking regions were extracted for all three cases homozygous for the *USH2A* c.2276G>T variant and the number of alleles carrying each SNV was determined. The majority of SNVs is shared amongst all alleles from chr1:216632415 (hg19, 35 kb upstream of *USH2A*) to chr1:216247667 (*USH2A* intron 27) (indicated with the red bar) or four alleles until chr1:216211989 (*USH2A* intron 32) (indicated with the orange bar).

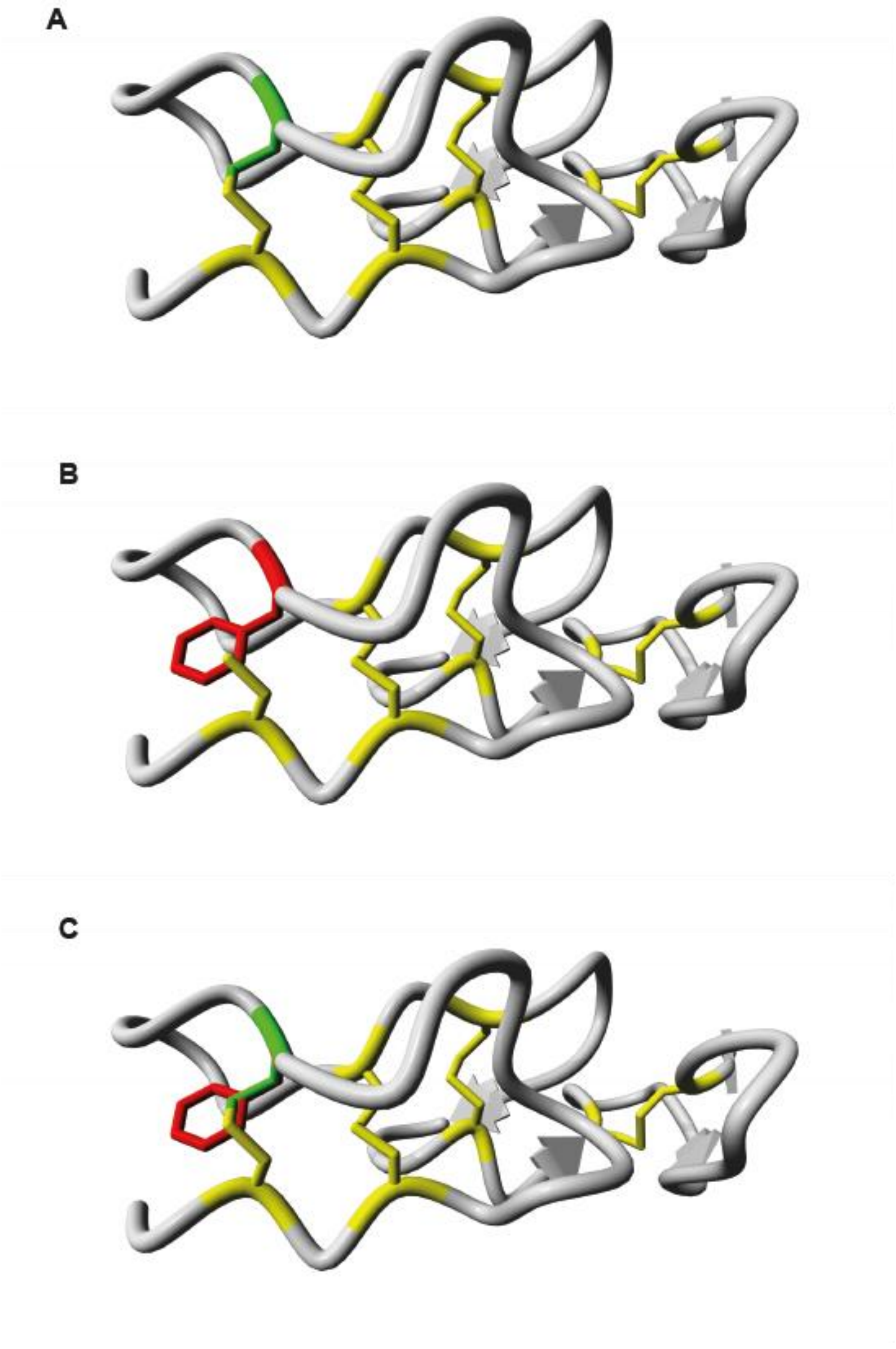

Supplementary Figure 2: ***In silico*** **modeling of the usherin fifth laminin–epidermal growth factor like domain.**

(A) p.(Cys759Phe) affects the fifth laminin–epidermal growth factor like domain of usherin. The cysteine residue at position 759 is depicted in green. Other cysteine residues and the disulfide bridges that are formed are depicted in yellow. (B) In red is the effect of the substitution of phenylalanine for cysteine. (C) A combined p.(Cys759Phe) model suggests the loss of a covalent cysteine bond which is detrimental for usherin folding and function.

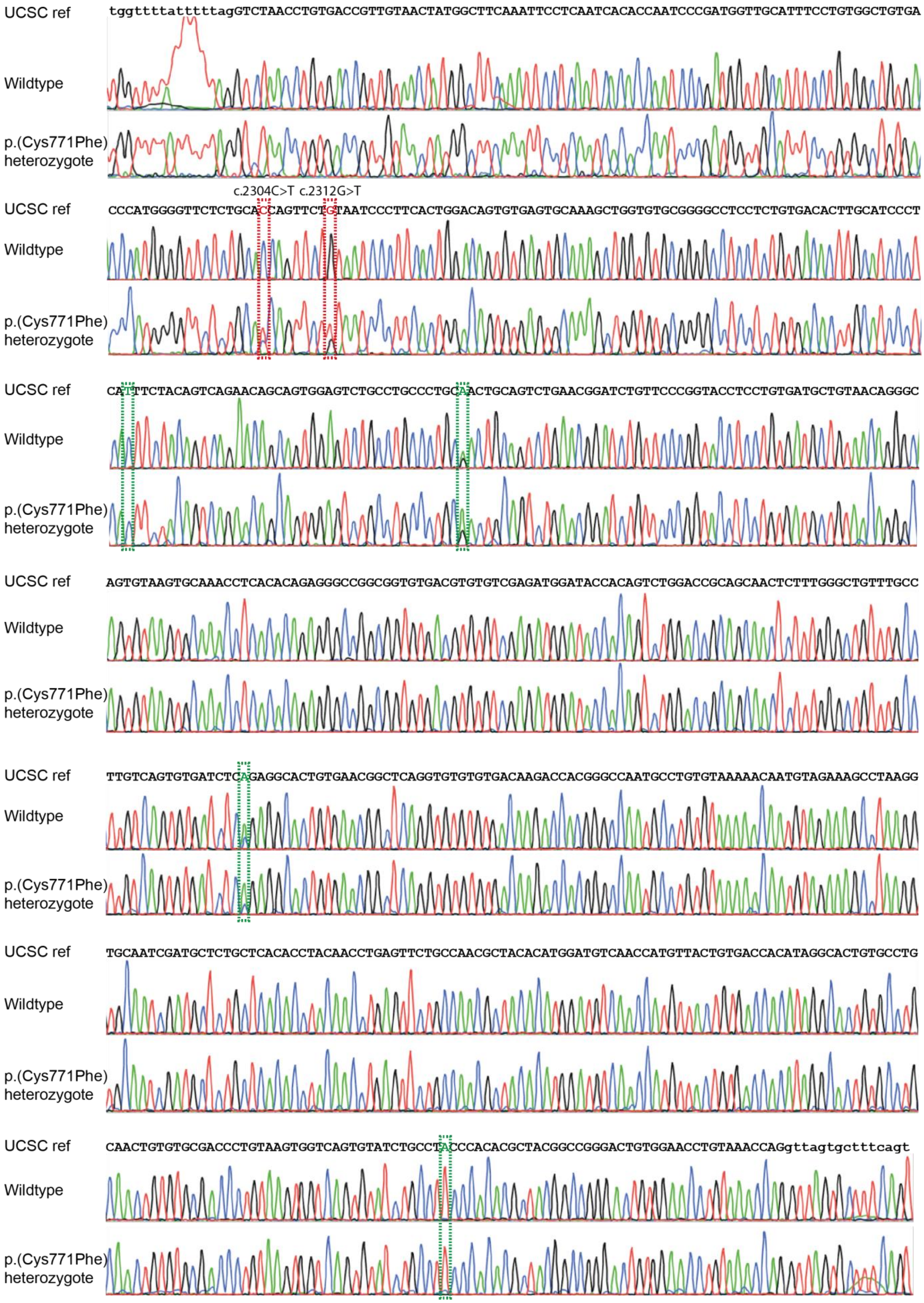

UCSC ref
Wildtype
p.(Cys771Phe) heterozygote
c.2304C>T
c.2312G>T

Supplementary Figure 3: Sanger sequencing traces of *ush2a* exon 13 and exon-intron boundaries. Sequence traces derived from genomic DNA of heterozygous p.(Cys771Phe) zebrafish (F1), strain-matched wildtypes and the UCSC reference sequence are shown. Variants c.2311G>T (p.(Cys771Phe)) and c.2304C>T (protospacer-adjacent motif-disrupting variant) are framed in red, deviations from the reference genome that were also identified in genomic DNA of wildtype zebrafish are framed in green. Nucleotides encoding exon 13 are in upper case, flanking intronic sequences in lower case. UCSC ref: UCSC reference genome (GRCz10/danRer10).

Supplementary Table 1: All single nucleotide variants in arRP-associated genes that met our pathogenicity criteria.

Sample zygosity	Gene name	Genomic position (hg19)	Variant	Protein effect	phyloP	CADD_ PHRED	Grantham Score	gnomAD-G AF (%)	SpliceAI _AG	SpliceAI _AL	SpliceAI _DG	SpliceAI _DL
I (het)	*ADGRA3*	4:g.22390410A>T	c.2884T>A	p.(Leu962Met)	0.1	23.1	15	0.01	0	0	0	0
I (het)	*CLCC1*	1:g.109479994A>G	c.725T>C	p.(Ile242Thr)	3.8	17.4	89	0.003	0	0	0.05	0
I (het)	*MVK*	12:g.110015065C>T	c.226+1115C>T		-0.6	0.1	0	0.1	0	0.04	0	0.12
I (het)	*NEK2*	1:g.211832090dup	c.1112-8dup		0.3	7.8	0	-	0	0	0	0
I (het)	*NEK2*	1:g.211832087_ 211832090dup	c.1112-11_ 1112-8dup		0.3	7.8	0	-	0	0	0	0
I (het)	*PDE6B*	4:g.646317G>C	c.712-1324G>C		-0.8	4.8	0	0.4	0	0	0	0.11
I (het)	*POMGNT1*	1:g.46664028C>G	c.-71G>C		-0.6	14.7	0	-	0	0	0.01	0.11
I (het)	*RP1*	8:g.55534671T>C	c.616-6T>C		1.3	15.8	0	0.4	0.01	0.26	0	0.24
I (het)	*RP1L1*	8:g.10480144G>A	c.568C>T	p.(Arg190Cys)	5.2	15.9	180	0.2	0	0.01	0	0
II (het)	*ABCA4*	1:g.94514466T>C	c.2701A>G	p.(Thr901Ala)	2.4	17.0	58	0.1	0	0.01	0	0
II (het)	*ARL6*	3:g.97485464A>T	c.-28+531A>T		-1.1	0.5	0	0.5	0.29	0	0.18	0
II (het)	*EYS*	6:g.65823605A>T	c.2024-55985T>A		-1.3	1.2	0	-	0	0.19	0	0.08
II (het)	*EYS*	6:g.65823607G>T	c.2024-55987C>A		0.5	3.3	0	-	0	0.21	0	0.09
II (het)	*FAM161A*	2:g.62069482G>A	c.197C>T	p.(Thr66Ile)	1.2	15.7	89	0.5	0	0	0	0
II (het)	*IFT172*	2:g.27677214A>G	c.3530+7T>C		0.0	10.6	0	0.1	0	0	0.02	0
II (het)	*RP1L1*	8:g.10480341G>A	c.371C>T	p.(Pro124Leu)	1.2	17.9	98	0.003	0	0	0	0
II (het)	*RPE65*	1:g.68906655T>C	c.524A>G	p.(Asn175Ser)	7.6	23.9	46	-	0.02	0.02	0	0.03
III (het)	*ARHGEF18*	19:g.7515874C>T	c.700-161C>T		-0.2	3.3	0	-	0	0	0.2	0.01
III (het)	*DHX38*	16:g.72135487C>T	c.1475C>T	p.(Thr492Met)	4.6	20.9	81	0.2	0.01	0	0	0
III (het)	*SLC7A14*	3:g.170244598G>A	c.128C>T	p.(Thr43Met)	3.8	19.5	81	-	0	0	0.01	0.01
All cases (hom)	*USH2A*	1:g.216420460C>A	c.2276G>T	p.(Cys759Phe)	5.5	28.9	205	0.1	0.03	0	0.01	0

No pathogenic homozygous or compound heterozygous single nucleotide variants, with the exception of *USH2A* c.2276G>T, could be observed in the three cases. The variants in *EYS* in case II are in *cis*. Variants in *RP1* and *RP1L1* are also associated with autosomal dominant RP, the variants in these genes identified in cases I and II are

classified as (likely) benign in ClinVar. GnomAD-G AF: Genome Aggregation Database: allele frequency in genome sequencing data, het: heterozygous, hom: homozygous.

Splice prediction scores are obtained with SpliceAI[1]. AG: acceptor gain, AL: acceptor loss, DG: donor gain, DL: donor loss.

Supplementary Table 2: Rare single nucleotide variants in *USH2A* that are shared amongst cases.

Variant (NM_206933.2)	Protein effect	Sample zygosity	phyloP	CADD_PHRED	Grantham Score	gnomAD-G AF (%)	SpliceAI_AG	SpliceAI_AL	SpliceAI_DG	SpliceAI_DL	SpliceSiteFinder-like (threshold: 75/100)	MaxEntScan (threshold AS: 12/16, DS: 9/12)	NNSPLICE (threshold: 0.75/1)	GeneSplicer (threshold AS: 15.75/21, DS: 18/24)
c.11712-206_11712-199dup		I (hom), II (hom), III (het)	0.6	1.7	0	-	na	na	na	na	-	-	-	-
c.6806-2881del		I (hom), II (het), III (hom)	-	2.5	0	-	0.04	0	0.03	0	-	-	-	-
c.4628-23020_4628-23007del		I (hom), II (hom), III (hom)	1.5	4.9	0	-	na	na	na	na	-	-	-	-
c.2276G>T	p.(Cys759Phe)	I (hom), II (hom), III (hom)	5.5	28.9	205	0.1	0.03	0	0.01	0	-	-	-	-
c.2256T>C	p.(His752=)	I (hom), II (het), III (hom)	-0.9	4.3	0	0.08	0.06	0.01	0.02	0	-	-	-	-
c.1971+7414dup		I (hom), II (het), III (hom)	-0.9	0.6	0	-	na	na	na	na	-	-	-	-
c.784+9428A>G		I (hom), II (hom), III (hom)	-0.8	4.0	0	0.1	0	0	0	0	Gain of AS: 79.4 to 79.6 (0.2%)	-	Gain of AS of 0.75 to 0.77 (2%)	-

GnomAD-G AF: Genome Aggregation Database: allele frequency in genome sequencing data, het: heterozygous, hom: homozygous. Splice prediction scores are obtained with SpliceAI[1], SpliceSiteFinder-like[2], MaxEntScan[3], NNSPLICE[4] and GeneSplicer[5]. AG: acceptor gain, AL: acceptor loss, DG: donor gain, DL: donor loss, AS: acceptor site, DS: donor site.

Supplementary Table 3: Genomic positions of putative *USH2A* regulatory regions and predicted *USH2A* promoter

#	Location (hg19)	Location (hg38)	Gene	Component	Human retina[6]	Mouse retina[7]	Mouse inner ear[8,9]	GeneHancer[10]	Cherry *et al.*[6]
1	chr1:216204347-216205162	chr1:216031005-216031820	*USH2A*	Intron 32	+	+	-	-	-
2	chr1:216228020-216228354	chr1:216054678-216055012		Intron 30	+	+	-	+	+
3	chr1:216263772-216264789	chr1:216090430-216091447		Intron 22	+	+	-	+	-
4	chr1:216394480-216394958	chr1:216221138-216221616		Intron 14	+	-	-	+	+
5	chr1:216408087-216408663	chr1:216234745-216235321		Intron 13	+	+	-	+	-
6	chr1:216560998-216562963	chr1:216387656-216389621		Intron 3	+	-	-	++	-
7*	chr1:216596228-216597072	chr1:216422886-216423730		Exon 1	+	+	-	+	+
8	chr1:216705232-216706130	chr1:216531890-216532788	*ESRRG*	Intron 7	+	+	-	+	+
9	chr1:216773974-216774910	chr1:216600632-216601568		Intron 5	+	-	-	+/-	+
10	chr1:216895153-216895595	chr1:216721811-216722253		Intron 3	+	+	-	+/-	+

* Region 7 is the predicted promoter of *USH2A*[6,10]. Data from human retina, mouse retina and mouse inner ear, was evaluated and each region was labeled with a '+' if a regulatory element was expected based on the specific data set. The GeneHancer database was assessed for enhancers (+) and whether they associate (++) or not (+/-) with the promoter of *USH2A*. Cherry *et al*. experimentally determined which regulatory regions were associated (+) or not (-) with the promoter of *USH2A*.

Supplementary Table 4: Single nucleotide variants in putative regulatory regions.

Gene name	Genomic location (hg19)	Variant	Sample zygosity	phyloP	CADD_PHRED	gnomAD-G AF (%)	SpliceAI_AG	SpliceAI_AL	SpliceAI_DG	SpliceAI_DL	SpliceSiteFinder-like (threshold: 75/100)	MaxEntScan (threshold AS: 12/16, DS: 9/12)	NNSPLICE (threshold: 0.75/1)	GeneSplicer (threshold AS: 15.75/21, DS: 18/24)
USH2A	1:g.216228343C>G	c.6050-6354G>C	I (hom), III (hom)	0.3	4.0	67.6	0	0	0	0	-	-	-	-
USH2A	1:g.216264294A>G	c.4759-1813T>C	I (hom), II (hom) III (hom)	1.2	16.9	51.5	0	0	0	0	-	-	-	-
USH2A	1:g.216408154G>A	c.2810-2676C>T	I (hom), II (hom) III (hom)	-0.5	2.3	66.5	0	0	0	0	-	-	-	-
USH2A	1:g.216561720G>A	c.652-23293C>T	I (hom), II (hom) III (hom)	-0.7	0.5	59.6	0	0	0	0	-	-	-	-
ESRRG	1:g.216705965T>C	c.794-13202A>G	II (het)	1.8	19.7	59.5	0	0	0	0	-	-	-	-
ESRRG	1:g.216774662G>A	c.521-33222C>T	I (hom), II (het), III (hom)	-0.4	2.8	15.0	0	0	0	0	Gain of AS: 73.8 to 76.9 (3.1%)	-	-	-
ESRRG	1:g.216895167T>C	c.-13-44334A>G	I (hom), II (hom) III (hom)	0.4	15.2	98.9	0	0	0	0.01	Loss of DS: 94.4 to 84.3 (−10.1%)	Loss of DS: 10.6 to 9.5 (−9.2%)	Loss of DS: 1 to 0.97 (−3%)	-

GnomAD-G AF: Genome Aggregation Database: allele frequency in genome sequencing data, het: heterozygous, hom: homozygous. Splice prediction scores are obtained with SpliceAI[1], SpliceSiteFinder-like[2], MaxEntScan[3], NNSPLICE[4] and GeneSplicer[5]. AG: acceptor gain, AL: acceptor loss, DG: donor gain, DL: donor loss, AS: acceptor site, DS: donor site.

Supplementary Table 5: Single nucleotide variants in *PDZD7*.

Variant (NM_ 001195263.1)	Protein effect	Sample zygosity	phyloP	CADD_ PHRED	Grantham Score	gnomAD-G AF (%)	SpliceAI _AG	SpliceAI _AL	SpliceAI _DG	SpliceAI _DL	SpliceSiteFinder-like (threshold: 75/100)	MaxEntScan (threshold AS: 12/16, DS: 9/12)	NNSPLICE (threshold: 0.75/1)	GeneSplicer (threshold AS: 15.75/21, DS: 18/24)
c.720-61T>G		I (het)	0.4	8.6	0	-	0.01	0	0.01	0	-	-	-	-
c.720-58A>G		I (het)	-0.3	6.2	0	-	0.01	0	0.01	0	-	-	-	-
c.720-56T>G		I (het)	-1.2	0.6	0	-	0.01	0	0	0	-	-	-	-
c.2325C>T	p.(Arg775=)	III (het)	0.2	18.1	0	0.01	na	na	na	na	-	-	-	-
c.2331T>C	p.(Arg777=)	III (het)	0.2	14.5	0	0.04	na	na	na	na	-	-	-	-
c.2618-250del		I (het)	-	1.3	0	-	na	na	na	na	-	-	-	-
c.2618-250dup		II (het)	-0.9	0.1	0	-	na	na	na	na	-	-	-	-
c.*587G>C		II (het)	0.3	3.3	0	0.7	na	na	na	na	-	-	-	-

GnomAD-G AF: Genome Aggregation Database: allele frequency in genome sequencing data, het: heterozygous, hom: homozygous. Splice prediction scores are obtained with SpliceAI[1], SpliceSiteFinder-like[2], MaxEntScan[3], NNSPLICE[4] and GeneSplicer[5]. AG: acceptor gain, AL: acceptor loss, DG: donor gain, DL: donor loss, AS: acceptor site, DS: donor site.

Supplementary Table 6: Primers used to amplify regions with potential unforeseen events due to CRISPR/Cas9 editing

Target	Forward primer	Reverse primer
ush2a exon 13 genomic analysis	CCAACAGAATCTAAATCTTTCTGGG	GCTGGCACATAACAAATAACAC
ush2a mRNA analysis (exons 11-13)	CCGTGCAGCCAGATATTACC	TGTATCTGCCTACCCACACG
chr5:40167334 (GRCz10)	ATTTAGCCGGTTAGCGTGTG	AGTCTTTGCCTTTGCCTGAT
chr10:44374669 (GRCz10)	Failed to obtain PCR product	
chr17:25767450 (GRCz10)	TGGCTGCCCTTAGTTAAAAC	AAGCATTAAAACACACAAAATGG
chr20:41755468 (GRCz10)	GCAATTTCAGAACTAGTACAGCC	CCGTCACTACTTCACACACG

Supplementary Table 7: Clinical data of three arRP cases, homozygous for *USH2A* c.2276G>T

Case	Sex	Ethnicity	Diagnosis	Age at diagnosis	Current age (2021)	Visual acuity (RE)	Visual acuity (LE)	Fundus	Autofluorescence	OCT	Visual field (Goldmann)	ERG
I	M	Dutch	Retinitis pigmentosa	32	43	2010: 0.7	2010: 0.8	Pallor of optic disc, attenuated vessels, bone spicules	NA	NA	2014: relative decrease of peripheral sensitivity	NA
II	F	Dutch	Retinitis pigmentosa	53	66	2017: 0.9 2019: S+1.25 C-0,50x75	2017: 0.8 2019: S+1.25 C-1.00x102	Waxy optic disc, dull macula, attenuated vessels, far-peripheral bone spicules	2017: hypoautofluorescent ring at the vascular arcades and surrounding the macular area	2017: loss of photoreceptor layers, with central residue.	2018: ring scotoma 15-35 degrees.	2017: decreased photopic and scotopic responses
III	M	Dutch	Retinitis pigmentosa	61	73	2008: 0,9 S 0.00 C-0.50 x95	2008: 0.7 (S+1.25 C-0.75x85)	2009: peripheral chorioretinal atrophy with residual preserved center	2008: chorioretinal atrophy	2017: central residue of photoreceptors	2008: tunnel vision (loss of peripheral vision, and preserved central vision) with peripheral residue superior and inferior	NA

ERG: electroretinogram, F: female, LE: left eye, M: male, NA: not analyzed, OCT: optical coherence tomography, RE: right eye

Supplementary Table 8: List of arRP-associated genes

ABCA4	*BEST1*	*CRB1*	*HGSNAT*	*MAK*	*PDE6B*	*RHO*	*SPATA7*
AGBL5	*C2orf71*	*CYP4V2*	*IDH3B*	*MERTK*	*PDE6G*	*RLBP1*	*TRNT1*
AHR	*C8orf37*	*DHDDS*	*IFT140*	*MVK*	*POMGNT1*	*RP1*	*TTC8*
ARHGEF18	*CERKL*	*DHX38*	*IFT172*	*NEK2*	*PRCD*	*RP1L1*	*TULP1*
ARL2BP	*CLCC1*	*EMC1*	*IMPG2*	*NEUROD1*	*PROM1*	*RPE65*	*USH2A*
ARL6	*CLRN1*	*EYS*	*KIAA1549*	*NR2E3*	*RBP3*	*SAG*	*ZNF408*
BBS1	*CNGA1*	*FAM161A*	*KIZ*	*NRL*	*REEP6*	*SAMD11*	*ZNF513*
BBS2	*CNGB1*	*GPR125 (ADGRA3)*	*LRAT*	*PDE6A*	*RGR*	*SLC7A14*	

Genes obtained from Retnet (visited May 21, 2021)[11].

Supplementary References

1 Jaganathan, K. *et al.* Predicting Splicing from Primary Sequence with Deep Learning. *Cell* **176**, 535-548.e524, doi:10.1016/j.cell.2018.12.015 (2019).

2 Shapiro, M. B. & Senapathy, P. RNA splice junctions of different classes of eukaryotes: sequence statistics and functional implications in gene expression. *Nucleic Acids Res* **15**, 7155-7174, doi:10.1093/nar/15.17.7155 (1987).

3 Yeo, G. & Burge, C. B. Maximum entropy modeling of short sequence motifs with applications to RNA splicing signals. *J Comput Biol* **11**, 377-394, doi:10.1089/1066527041410418 (2004).

4 Reese, M. G., Eeckman, F. H., Kulp, D. & Haussler, D. Improved splice site detection in Genie. *J Comput Biol* **4**, 311-323, doi:10.1089/cmb.1997.4.311 (1997).

5 Pertea, M., Lin, X. & Salzberg, S. L. GeneSplicer: a new computational method for splice site prediction. *Nucleic Acids Res* **29**, 1185-1190, doi:10.1093/nar/29.5.1185 (2001).

6 Cherry, T. J. *et al.* Mapping the cis-regulatory architecture of the human retina reveals noncoding genetic variation in disease. *Proc Natl Acad Sci U S A* **117**, 9001-9012, doi:10.1073/pnas.1922501117 (2020).

7 Mo, A. *et al.* Epigenomic landscapes of retinal rods and cones. *Elife* **5**, e11613, doi:10.7554/eLife.11613 (2016).

8 Muthu, V. *et al.* Genomic architecture of Shh-dependent cochlear morphogenesis. *Development* **146**, doi:10.1242/dev.181339 (2019).

9 Yizhar-Barnea, O. *et al.* DNA methylation dynamics during embryonic development and postnatal maturation of the mouse auditory sensory epithelium. *Sci Rep* **8**, 17348, doi:10.1038/s41598-018-35587-x (2018).

10 Fishilevich, S. *et al.* GeneHancer: genome-wide integration of enhancers and target genes in GeneCards. *Database (Oxford)* **2017**, doi:10.1093/database/bax028 (2017).

11 Daiger, S. R., BJF.; Greenberg, J.; Christoffels, A.; Hide, W. *RetNet, the Retinal Information Network*, <https://sph.uth.edu/RetNet/> (1998).
