# Supplementary material for: Scrutinizing pathogenicity of the USH2A c.2276 G > T; p.(Cys759Phe) variant
Source: NPJ Genom Med. 2022 Jun 7;7:37. doi: 10.1038/s41525-022-00306-z (PMC9174243; doi:10.1038/s41525-022-00306-z)
Supplement: Supplementary file 2 — Reporting Summary Checklist [file 41525_2022_306_MOESM2_ESM.pdf]

nature portfolio

Corresponding author(s): Erwin van Wijk; NPJGENMED-01384

Last updated by author(s): Apr 19, 2022

Reporting Summary

Nature Portfolio wishes to improve the reproducibility of the work that we publish. This form provides structure for consistency and transparency in reporting. For further information on Nature Portfolio policies, see our Editorial Policies and the Editorial Policy Checklist.

Statistics

For all statistical analyses, confirm that the following items are present in the figure legend, table legend, main text, or Methods section.

n/a	Confirmed	
☐	☒	The exact sample size (*n*) for each experimental group/condition, given as a discrete number and unit of measurement
☐	☒	A statement on whether measurements were taken from distinct samples or whether the same sample was measured repeatedly
☐	☒	The statistical test(s) used AND whether they are one- or two-sided<br>*Only common tests should be described solely by name; describe more complex techniques in the Methods section.*
☒	☐	A description of all covariates tested
☐	☒	A description of any assumptions or corrections, such as tests of normality and adjustment for multiple comparisons
☐	☒	A full description of the statistical parameters including central tendency (e.g. means) or other basic estimates (e.g. regression coefficient) AND variation (e.g. standard deviation) or associated estimates of uncertainty (e.g. confidence intervals)
☐	☒	For null hypothesis testing, the test statistic (e.g. *F*, *t*, *r*) with confidence intervals, effect sizes, degrees of freedom and *P* value noted<br>*Give P values as exact values whenever suitable.*
☒	☐	For Bayesian analysis, information on the choice of priors and Markov chain Monte Carlo settings
☒	☐	For hierarchical and complex designs, identification of the appropriate level for tests and full reporting of outcomes
☒	☐	Estimates of effect sizes (e.g. Cohen's *d*, Pearson's *r*), indicating how they were calculated

Our web collection on statistics for biologists contains articles on many of the points above.

Software and code

Policy information about availability of computer code

Data collection	n/a
Data analysis	Whole genome sequencing data processing and analysis was performed as described in the methods section. Splice predictions were obtained from SpliceAI (https://github.com/Illumina/SpliceAI) and the splice prediction tools that are freely available Alamut Visual (version 2.13): SpliceSiteFinder-like, MaxEntScan, NNSPLICE and GeneSplicer. Screening for off-target effects was performed with Cas-OFFinder (http://www.rgenome.net/cas-offinder/). Immunohistochemistry images were processed with ZEN software (version 3.1 (blue edition)) and quantified with Fiji (version 1.51n). All statistical analyses were performed using PRISM software (v9.0.0).

For manuscripts utilizing custom algorithms or software that are central to the research but not yet described in published literature, software must be made available to editors and reviewers. We strongly encourage code deposition in a community repository (e.g. GitHub). See the Nature Portfolio guidelines for submitting code & software for further information.

Data

Policy information about availability of data

All manuscripts must include a data availability statement. This statement should provide the following information, where applicable:

- Accession codes, unique identifiers, or web links for publicly available datasets
- A description of any restrictions on data availability
- For clinical datasets or third party data, please ensure that the statement adheres to our policy

Data are available upon request. All whole genome sequencing variants that were considered to be potentially pathogenic are available in the supplementary data.

Field-specific reporting

Please select the one below that is the best fit for your research. If you are not sure, read the appropriate sections before making your selection.

☒ Life sciences ☐ Behavioural & social sciences ☐ Ecological, evolutionary & environmental sciences

For a reference copy of the document with all sections, see nature.com/documents/nr-reporting-summary-flat.pdf

Life sciences study design

All studies must disclose on these points even when the disclosure is negative.

Sample size	- Whole genome sequencing was performed for three cases homozygous for USH2A c.2276G>T (p.(Cys759Phe)) of whom DNA was available.<br>- Samples sizes of ERG measurements are based on experience with regard to previous zebrafish models for USH2A-associated disease. In addition, we performed a sample size calculation based on an expected reduction in B wave amplitude of 15% (https://clincalc.com/stats/samplesize.aspx) of two independent groups with continuous measurement; mean of group 1: 1 ± 0.2; mean of group 2: 0.85; alpha: 0.05; power: 80%; resulting in a minimum of 28 samples for each group.<br>- Sample sizes of immunohistochemistry for usherin and rhodopsin are based on experience with regard to previous zebrafish models for USH2A-associated disease; we considered a samples size of 6 - 14 larval eyes per group sufficient to observe a potential significant effect.
Data exclusions	- ERGs were recorded on a blinded sample set: if the shape the B wave was considered abnormal, the measurement was excluded.<br>- Images for quantification of aberrant rhodopsin localization were scored by two individual researchers. Images were excluded in case of insufficient quality, defined by inappropriate cross-section, incomplete eyes, and poor morphology.
Replication	- Three biological replicate experiments were performed for ERG measurements.<br>- Immunohistochemical analyses using anti-usherin and anti-rhodopsin antibodies using different ush2a zebrafish models have been repeatedly published before with similar outcomes (as described in the manuscript). Results in the current manuscript are comparable with the previously published data and therefore considered reproducible.
Randomization	- Randomization of human research participants was not relevant in our study.<br>- Randomization of immunohistochemical images for usherin staining was not relevant as these images were scored by an automated script.<br>- Randomization of immunohistochemical images for quantification of aberrant rhodopsin localization was performed before the images were scored by two independent researchers.<br>- For ERG measurements randomization of larvae was not feasible and therefore no randomization was performed. Instead, blinded samples containing either wildtype or mutant larvae were recorded alternately.
Blinding	- Personalia of human research participants were blinded to the researchers, except for the lead investigator and clinicians.<br>- Blinding of immunohistochemical images for usherin staining was not relevant as these images were scored by an automated script.<br>- Blinding of immunohistochemical images for quantification of aberrant rhodopsin localization was performed before the images were scored by two independent researchers.<br>- For ERG measurements blinding was performed by random labeling of both groups (wildtype and mutant) of larvae before they were handed over to an independent researcher that performed the measurements.

Reporting for specific materials, systems and methods

We require information from authors about some types of materials, experimental systems and methods used in many studies. Here, indicate whether each material, system or method listed is relevant to your study. If you are not sure if a list item applies to your research, read the appropriate section before selecting a response.

Materials & experimental systems

n/a	Involved in the study	
☐	☒	Antibodies
☐	☒	Eukaryotic cell lines
☒	☐	Palaeontology and archaeology
☐	☒	Animals and other organisms
☐	☒	Human research participants
☒	☐	Clinical data
☒	☐	Dual use research of concern

Methods

n/a	Involved in the study	
☒	☐	ChIP-seq
☒	☐	Flow cytometry
☒	☐	MRI-based neuroimaging

Antibodies

Antibodies used	Usherin:<br>Primary antibody: rabbit anti-usherin-C (1:500, #27640002, Lot: 0011912DZ5381524, Novus Biological, Centennial (CO), USA)<br>Secondary antibody: goat anti-rabbit Alexa Fluor® 568 (1:800, A11011, Lot: 2277758, Molecular Probes, Eugene (OR), USA)

Centrin:
Primary antibody: mouse anti-centrin (1:500, #04-1624, Lot: 2829820, Millipore, Burlington (MA), USA)
Secondary antibody: goat anti-mouse Alexa Fluor® 488 (1:800, A11029, Lot: 2277759, Molecular Probes, Eugene (OR), USA)

Rhodopsin:
Primary antibody: mouse anti-rhodopsin (1:2000, Clone 4D2, NBP2-59690, Lot: MR187076, Novus Biological, Centennial (CO), USA)
Secondary antibody: goat anti-mouse Alexa Fluor® 488 (1:800, A11029, Lot: 2179204, Molecular Probes, Eugene (OR), USA)

Validation

Employment and validation of all antibodies described above has been published previously in Dulla et al. (PMID: 33895329), Toms et al. (PMID: 31998945), Slijkerman et al. (PMID: 30281416) and Corral-Serrano et al. (PMID: 29946172).

Eukaryotic cell lines

Policy information about cell lines

Cell line source(s)	HEK293T
Authentication	Purchased from ATTC (https://www.atcc.org/products/crl-3216)
Mycoplasma contamination	Cell lines were tested for mycoplasma contamination
Commonly misidentified lines (See ICLAC register)	n/a

Animals and other organisms

Policy information about studies involving animals; ARRIVE guidelines recommended for reporting animal research

Laboratory animals	Zebrafish (Danio Rerio) were used; Strain: Tüpfel long fin (https://zfin.org/ZDB-GENO-990623-2); Sex: Mixed; Age: 5 days post fertilization
Wild animals	Study did not involve wild animals
Field-collected samples	Study did not involve samples collected in the field
Ethics oversight	The animal experiments were approved by the Radboud University Institutional Review Board of the Centrale Commissie Dierproeven (AVD103002017945).

Note that full information on the approval of the study protocol must also be provided in the manuscript.

Human research participants

Policy information about studies involving human research participants

Population characteristics	Three cases with autosomal recessive retinitis pigmentosa that are homozygous for USH2A c.2276G>T (p.(Cys759Phe)) were included.
Recruitment	All cases were recruited from the Radboud University Medical Center Nijmegen and were included because they remained genetically unexplained after diagnostic testing.
Ethics oversight	The study protocol was approved by the local ethics committee of the Radboud University Medical Center Nijmegen, as an amendment to the approval by the local ethics committee of the Rotterdam Eye Hospital (MEC-2010-359; OZR protocol no. 2009-32).

Note that full information on the approval of the study protocol must also be provided in the manuscript.
